# Systemic medications associate with surgically treated cataract among adults over 50 years in Finland

**DOI:** 10.1111/aos.70118

**Published:** 2026-03-11

**Authors:** Antti Riikonen, Jari Haukka, Tuomas Lilius, Sirpa Loukovaara

**Affiliations:** ^1^ Unit of Vitreoretinal Surgery, Department of Ophthalmology Helsinki University Hospital Helsinki Finland; ^2^ Department of Public Health University of Helsinki Helsinki Finland; ^3^ Individualized Drug Therapy Research Program University of Helsinki Helsinki Finland; ^4^ Department of Emergency Medicine and Services HUS Helsinki University Hospital and University of Helsinki Helsinki Finland; ^5^ Division of Pharmacology and Pharmacotherapy University of Helsinki Finland

**Keywords:** association, cataract surgery, exposure, nested case–control, real world evidence, systemic medication

## Abstract

**Purpose:**

To identify associations between systemic drugs and cataract surgery in Finland.

**Methods:**

A historic cohort study based on administrative data. Endpoint event was cataract surgery. Use of drugs in question was based on redeemed prescriptions and consisted of 156 drugs. Study population consisted of 106 127 individuals aged ≥50 years without diabetes originating from the 1996–2017 Fin‐CARING population. We constructed a nested case–control study design comparing 17 811 cases undergoing cataract surgery to 76 532 age‐, sex‐ and hospital district‐matched controls without cataract surgery. Results are reported as odds ratios (ORs) with 95% confidence intervals (CIs) based on conditional logistic regression models.

**Results:**

Use of ramipril (1.14; 1.04–1.25), losartan (1.20; 1.07–1.35), bisoprolol (1.24; 1.18–1.31), amlodipine (1.16; 1.07–1.25), diltiazem (1.26; 1.06–1.49), isosorbide mononitrate (1.56; 1.44–1.70), furosemide (1.31; 1.18–1.45), simvastatin (1.17; 1.11–1.24), atorvastatin (1.17; 1.06–1.29), prednisolone (2.00; 1.64–2.42), allopurinol (1.47; 1.24–1.75), pramipexole (1.45; 1.13–1.86), testosterone (1.62; 1.03–2.55), estradiol (1.13; 1.03–1.24), oxazepam (1.26; 1.07–1.47), sertraline (1.74; 1.13–2.67) and zopiclone (1.31; 1.20–1.43) showed some evidence for an association with cataract surgery. Use of memantine (OR 0.24; 0.15–0.40), risperidone (0.72; 0.51–1.00), rivastigmine (0.35; 0.21–0.58) and donepezil (0.55; 0.40–0.76) was associated with a lower risk of cataract surgery.

**Conclusions:**

Our large nested case–control study suggests that certain systemic medications may increase the incidence of surgically treated cataract, but with the possibility of residual confounding.

## 1 INTRODUCTION

Cataract is a major cause of treatable blindness worldwide. Pathogenesis of age‐related cataract is complex, multifactorial, induced by numerous genetic, epigenetic and environmental sources. Molecular mechanisms such as oxidative stress and over‐production of reactive oxygen species have been implicated as underlying causes of cataracts (Fang, Huang, et al., 2019; Fang, Ye, et al., 2019; Truscott, 2005). Although ageing is the leading cause of age‐related cataract, there are multiple factors, such as smoking, diabetes, sunlight exposure (ultraviolet B) and ionizing radiation that are known to be associated with the development of age‐related cataract (Klein et al., 2003; Schueler & Fetterly, 2021; Stefek & Karasu, 2011). Noteworthy, adherence to a healthy diet including vitamins, antioxidants, fruits and vegetables is known to be associated with a lower risk of age‐related cataracts (Zhao et al., 2014; Zhou et al., 2022).

Epidemiological studies have consistently found that post‐menopausal women have a higher risk of cataract than age‐matched men (Zetterberg & Celojevic, 2015). It has also been known for decades that the incidence of cataract surgery is higher in older women compared to men of the same age (Lundstrom et al. 1999; Faranda et al., 2021). Previous epidemiological studies have shown that the need for cataract surgery is significantly more likely associated with positive histories for diabetes mellitus, hypertension, dyslipidaemia, cardiovascular and respiratory diseases (Erie et al., 2016).

However, less is known about systemic medications used for the common age‐related systemic diseases. Since long the use of corticosteroids has been established as a risk factor for the development of posterior subcapsular cataract (Urban Jr & Cotlier, 1986). Associations of other systemic medications, including statins, antihypertensive drugs and antidepressants, have been more controversial. Recently, it was shown that especially tricyclic antidepressants, insulin and some antiarrhythmic agents could be associated with surgically treated cataract (Deng et al., 2023). That study emphasized the need to evaluate further the role of less known risk factors such as systemic drugs and to intervene on the modifiable risks to decrease the incidence of cataract surgery (Deng et al., 2023). Another paper showed an association with cortical cataract and the use of angiotensin‐converting enzyme (ACE) inhibitors, fibrates, alpha‐glucosidase inhibitors and insulin (Dai et al., 2020).

Today, large‐scale studies examining the incidence of cataract surgery associated with the use of systemic medications are lacking. The main aim of our retrospective longitudinal cohort study was to compare commonly used systemic medications, including antihypertensive drugs, cardiovascular agents, statins, hormones, metabolic agents and central nervous system agents with respect to the development of age‐related cataract leading to surgical cataract removal in a large real‐world cohort in Finland.

Topic of the study was considered important since there is a persistent need to better understand the development of age‐related cataract (*cataractogenesis*) among different populations and ethnic backgrounds in the world.

## 2 METHODS

Our study population consisted of a subpopulation derived from the Fin‐CARING project, as detailed previously (Niskanen et al., 2020). Briefly, the original CARING cohort (*n* = 398 708) contained 199 354 subjects with diabetes mellitus (DM) and an equivalent reference population matched by age, sex and hospital district.

### 2.1 Data collection and analysis

The data were extracted from administrative healthcare registers including Social Insurance Institution, The Finnish Institute for Health and Welfare, the Finnish Cancer Registry and Statistics Finland (Loukovaara et al. 2022). Socio‐economic data were retrieved from Statistics Finland, and socio‐economic group variables are defined according to their Classification of Socio‐economic Groups 1989 (https://stat.fi/en/luokitukset/sosioekon_asema/sosioekon_asema_1_19890101).

Our study population contained individuals who had purchased and received reimbursement between 1 January 1996 and 31 December 2017, for at least two prescriptions per year of the systemic medications of interest for a minimum of 5 years before developing surgically operated cataract in Finland (Niskanen et al., 2020). In Finland, patients are reimbursed for filled prescriptions up to 3 months at a time. This means that in the registers, a continuously used drug shows as several purchased and reimbursed prescriptions.

We investigated associations between use of commonly prescribed systemic medications and incidence of cataract surgery in the Finnish population with long‐term exposure to 210 systemic drug substances and 45 combinations of those.

### 2.2 Study population

**FIGURE 1:**
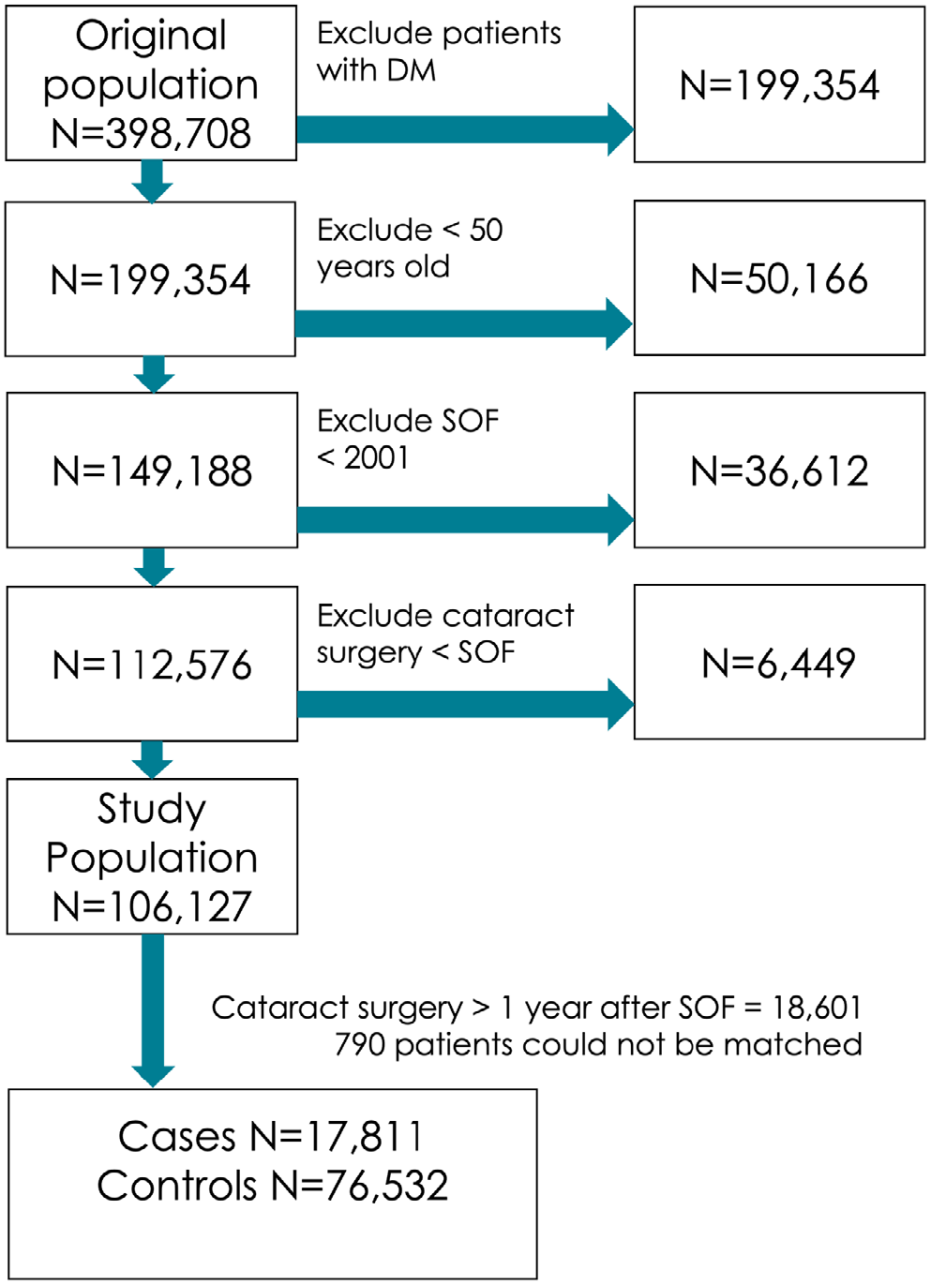
Flow diagram describes the selection of the nested case‐control study population out of the 106,127 individuals without diabetes. The exclusion of cataract surgery is due to the washout period. DM, diabetes mellitus; SOF, start of follow‐up.

Our original study population consisted of 398 708 individuals, out of which a total of 199 354 individuals had diabetes and were excluded (Figure 1). Patients with diabetes were excluded since diabetes is known to cause cataracts, as shown in multiple studies, including The Blue Mountain Eye Study (Tan et al., 2008).

Finally, we included 106 127 persons who did not have diabetes, were at least 50 years old in the start of follow‐up, who did not have cataract surgery before start of follow‐up (SOF) and whose follow‐up started after 2000.

### 2.3 Surgically treated cataract

Surgically treated cataract was defined with a surgical procedure code for phacoemulsification cataract surgery, CJE20 (NOMESCO classification of surgical procedures 2010) (Nordisk Medicinal Statistik Komite, 2010). Other cataract procedures, such as manual small incision cataract surgery, are exceedingly rare in Finland and were not included in our study. The outcome was first eye cataract procedure between 1 January 2000 and 31 December 2017. Among our study population, we observed altogether 18 601 cataract procedures (by phacoemulsification).

### 2.4 Nested case–control study design

Based on the incidence data, we constructed a nested case–control (NCC) study by matching each person undergoing cataract surgery (*n* = 17 811 cases) with five age‐ (1‐year calibre), sex‐ and hospital district adjusted controls who had not undergone cataract surgery until the time of outcome event (*n* = 76 532 controls) (Figure 1, Figure S1). In the nested case–control study, incidence density sampling is used by default, with replacement. Matching could not be done for 790 cases, and 5065 case–control sets were incomplete.

### 2.5 Exposure to systemic drug classes

We used the number of redeemed prescriptions in 5 years before the event (i.e. cataract surgery) as exposures. We calculated the exposure to systemic medication for a nested case–control design in two alternative ways:

1. Indicator (0/1) for having purchased at least two prescriptions per year within a 5‐year period preceding the index cataract operation.
2. A continuous variable was generated to estimate the trend based on the number of years with at least two prescriptions per year within a 5‐year period preceding the index cataract operation.

### 2.6 Ethics

This study was conducted according to the principles of the Declaration of Helsinki. The research ethics committee at the Faculty of Medicine, University of Helsinki gave the study its approval on 17 January 2012 (Ref 02/2012). The reimbursement prescription data were received from the Social Insurance Institution (SII) (permission Kela 16/522/2012) that approved the extension of the study plan and research group (29/522/2019). The procedure codes were received from The Finnish Institute for Health and Welfare (THL, 486/5.05.00/2019, Joona Klemetti). All data processing practices followed the EU Data Protection Directive rules. Informed consent was not required due to the register‐based study design.

### 2.7 Statistical analysis

Nested case–control (NCC) design was analysed with conditional logistic regression that takes matching into account. Two models with both exposures were applied: (1) no covariates and (2) socio‐economic group as a covariate.

Results are reported as odds rations (OR) with 95% confidence intervals (CIs), and q‐values. To control false discovery rate (FDR), we report q‐values instead of p‐values (Benjamini & Hochberg, 1995; Storey et al., 2004; Storey et al., 2024). In brief, ‘q‐value’ is the FDR‐based measure of significance.

The data were analysed using the R language (R Core Team, 2019).

## 3 RESULTS

### 3.1 Baseline characteristics

A total of 94 343 participants were included in the study population, out of which 17 811 underwent cataract surgery and 76 532 were age‐ and sex‐matched references. The mean age was 76.2 (SD 7.4) years among the surgical group compared to 75.4 (SD 7.3) years in the reference group. More than 57% of the individuals were female in both groups (*p* = 0.46) (Table 1). Socio‐economically, the groups were balanced.

**TABLE 1:** Basic characteristics of individuals without diabetes who underwent cataract surgery (CJE20) (*n* = 17 811) compared with those who did not receive surgery (*n* = 76 532).

	Overall	Controls	Cases
*n*	94 343	76 532	17 811
Age (mean (SD))	75.52 (7.3)	75.38 (7.3)	76.17 (7.4)
Female (%)	54 100 (57.3)	43 842 (57.3)	10 258 (57.6)
Socio‐economic background (%)^a^			
Upper‐level employees	3869 (4.1)	3236 (4.2)	633 (3.6)
Self‐employed	3001 (3.2)	2548 (3.3)	270 (10.3)
Lower‐level employees	5602 (5.9)	4682 (6.1)	920 (5.2)
Manual workers	5932 (6.3)	4921 (6.4)	1011 (5.7)
Students	235 (0.2)	195 (0.3)	40 (0.2)
Pensioners	71 475 (75.8)	57 442 (75.1)	14 033 (78.8)
Others	4229 (4.5)	3508 (4.6)	721 (4.0)

^a^ Based on the data from Statistics Finland.

### 3.2 Associations of systemic drugs with surgically treated cataract

We identified several drugs that were significantly associated with surgical removal of age‐related cataract.

According to the analysis, when we considered two redeemed prescriptions per year in 5 years before the event, exposure to use of ACE inhibitor ramipril (1.14; 1.04–1.25), angiotensin II receptor (AT2R) antagonist losartan (1.20; 1.07–1.35), beta blocker bisoprolol (1.24; 1.18–1.31), calcium channel blockers amlodipine (1.16; 1.07–1.25) and diltiazem (1.26; 1.06–1.49), cardiovascular agents isosorbide mononitrate (1.56; 1.44–1.70) and isosorbide dinitrate (1.36; 1.21–1.53), diuretic furosemide (1.31; 1.18–1.45), statins simvastatin (1.17; 1.11–1.24) and atorvastatin (1.17; 1.06–1.29), corticosteroid prednisolone (2.00; 1.64–2.42), metabolic agent allopurinol (1.47; 1.24–1.75), dopamine agonist pramipexole (1.45; 1.13–1.86), sex hormones testosterone (1.62; 1.03–2.55) and estradiol (1.13; 1.03–1.24), benzodiazepine oxazepam (1.26; 1.07–1.47), antidepressant sertraline (1.74; 1.13–2.67) as well as insomnia medication zopiclone (1.31; 1.20–1.43) was significantly associated with surgically treated cataract as presented in Table 2.

**TABLE 2:** Exposure to systemic medications with at least two redeemed prescriptions every year for 5 years before cataract surgery event in persons without diabetes compared to their matched controls who did not undergo surgery.

ATC	N.Control	N.Case	OR.Unadj	*q*‐value.Unadj	OR.Adj	*q*‐value.Adj
C01AA05 (digoxin)	561	177	1.25 (1.05–1.49)	0.01535325	1.24 (1.05–1.48)	0.01898125
C01BC04 (flecainide)	206	60	1.28 (0.96–1.71)	0.07119913	1.28 (0.96–1.71)	0.07827260
C01DA02 (glyceryl trinitrate)	250	83	1.21 (0.93–1.56)	0.10418816	1.20 (0.93–1.56)	0.11270303
C01DA08 (isosorbide dinitrate)	1136	394	1.36 (1.21–1.53)	0.00000218	1.35 (1.20–1.52)	0.00000369
C01DA14 (isosorbide mononitrate)	2336	892	1.56 (1.44–1.70)	0.00000000	1.55 (1.43–1.68)	0.00000000
C02AC05 (moxonidine)	163	57	1.43 (1.05–1.94)	0.02332659	1.43 (1.05–1.94)	0.02580555
C03AA03 (hydrochlorodiazide)	389	105	1.18 (0.95–1.47)	0.09273521	1.18 (0.95–1.47)	0.10406358
C03BA11 (indapamide)	116	35	1.29 (0.88–1.89)	0.11788653	1.29 (0.88–1.89)	0.13271776
C03CA01 (furosemide)	1655	553	1.31 (1.18–1.45)	0.00000088	1.30 (1.18–1.44)	0.00000191
C03DA01 (spironolactone)	358	99	1.17 (0.93–1.47)	0.11070378	1.16 (0.92–1.45)	0.14080005
C03EA01 (hydrochorodiatzide and potassium‐sparing agents)	1843	452	1.03 (0.92–1.14)	0.26329148	1.02 (0.92–1.14)	0.29924850
C03EA02 (trichlormethiazide and potassium‐sparing agents)	213	71	1.45 (1.11–1.91)	0.01068648	1.46 (1.11–1.92)	0.01094865
C03EB01 (furosemide and potassium‐sparing agents)	271	106	1.52 (1.20–1.91)	0.00095601	1.51 (1.20–1.90)	0.00124348
C07AA03 (pindolol)	135	35	1.06 (0.72–1.54)	0.29120875	1.06 (0.73–1.55)	0.32413210
C07AA05 (propranolol)	306	82	1.14 (0.89–1.46)	0.16453051	1.11 (0.87–1.42)	0.22196040
C07AA07 (sotalol)	242	77	1.23 (0.94–1.60)	0.08973125	1.22 (0.93–1.58)	0.10696330
C07AB02 (metoprolol)	5248	1402	1.11 (1.04–1.18)	0.00257863	1.10 (1.04–1.18)	0.00423016
C07AB03 (atenolol)	801	241	1.29 (1.11–1.49)	0.00164719	1.28 (1.11–1.48)	0.00232730
C07AB04 (acebutolol)	203	58	1.16 (0.86–1.56)	0.17090937	1.15 (0.85–1.55)	0.20537980
C07AB05 (betaxolol)	329	85	1.08 (0.85–1.38)	0.22928694	1.08 (0.84–1.37)	0.26617573
C07AB07 (bisoprolol)	7280	2104	1.24 (1.18–1.31)	0.00000000	1.24 (1.17–1.30)	0.00000000
C07AB08 (celiprolol)	279	88	1.36 (1.07–1.74)	0.01535325	1.35 (1.06–1.72)	0.02004004
C07AG02 (carvedilol)	683	162	1.01 (0.85–1.21)	0.31863949	1.00 (0.84–1.20)	0.37779733
C07BB02 (metoprolol and thiazides)	176	51	1.25 (0.91–1.71)	0.11070378	1.24 (0.90–1.70)	0.12749555
C07BB07 (bisoprolol and thiazides)	534	133	1.04 (0.86–1.264)	0.26941244	1.03 (0.85–1.26)	0.31644658
C07FB02 (metoprolol and felodipine)	458	103	0.94 (0.76–1.17)	0.24084290	0.94 (0.76–1.17)	0.26617573
C08CA01 (amlodipine)	3314	905	1.16 (1.07–1.25)	0.00050622	1.16 (1.07–1.25)	0.00078666
C08CA02 (felodipine)	1396	378	1.14 (1.02–1.29)	0.02332659	1.14 (1.02–1.28)	0.02737803
C08CA03 (isradipine)	129	25	0.85 (0.55–1.31)	0.21172388	0.85 (0.55–1.31)	0.23838549
C08CA05 (nifedipine)	573	167	1.24 (1.04–1.48)	0.01934287	1.23 (1.03–1.47)	0.02422950
C08CA07 (nisoldipine)	89	25	1.09 (0.68–1.72)	0.28158437	1.09 (0.68–1.72)	0.31644658
C08CA10 (nilvadipine)	173	53	1.28 (0.93–1.75)	0.08973125	1.26 (0.92–1.73)	0.10647192
C08CA13 (lercandipine)	1003	283	1.18 (1.03–1.35)	0.01943735	1.17 (1.02–1.34)	0.02422950
C08DA01 (verapamil)	237	49	0.84 (0.62–1.15)	0.15687354	0.84 (0.61–1.15)	0.17122165
C08DB01 (diltiazem)	608	182	1.26 (1.06–1.49)	0.01071805	1.25 (1.06–1.48)	0.01398764
C09AA01 (captopril)	137	48	1.45 (1.04–2.02)	0.02707965	1.44 (1.03–2.02)	0.03270612
C09AA02 (enalapril)	2344	606	1.10 (0.99–1.20)	0.04107352	1.09 (0.99–1.19)	0.07321678
C09AA03 (lisinopril)	446	106	1.04 (0.84–1.28)	0.28650570	1.04 (0.84–1.29)	0.31644658
C09AA04 (perindopril)	347	108	1.35 (1.08–1.68)	0.01068648	1.35 (1.08–1.68)	0.01256433
C09AA05 (ramipril)	2317	615	1.14 (1.04–1.25)	0.00700937	1.14 (1.04–1.25)	0.01026741
C09BA02 (enalapril and diuretics)	1264	286	0.99 (0.87–1.13)	0.31262281	0.98 (0.86–1.12)	0.32860991
C09BA03 (lisinopril and diuretics)	274	61	0.93 (0.70–1.23)	0.24888674	0.93 (0.70–1.23)	0.28486861
C09BA05 (ramipril and diuretics)	95	27	1.27 (0.82–1.95)	0.15749425	1.26 (0.82–1.95)	0.18252623
C09CA (AT2R antagonists)	3890	1066	1.15 (1.07–1.24)	0.00040902	1.15 (1.07–1.23)	0.00050924
C09CA01 (losartan)	1391	398	1.20 (1.07–1.35)	0.00342732	1.20 (1.07–1.34)	0.00440433
C09CA02 (eprosartan)	99	19	0.81 (0.49–1.33)	0.19232769	0.80 (0.49–1.33)	0.21456951
C09CA03 (valsartan)	563	144	1.06 (0.88–1.28)	0.23773316	1.06 (0.88–1.27)	0.26617573
C09CA06 (candesartan)	1310	356	1.15 (1.02–1.29)	0.02350143	1.15 (1.02–1.30)	0.02580555
C09CA07 (telmisartan)	326	84	1.06 (0.83–1.35)	0.26389214	1.06 (0.83–1.35)	0.29924850
C09DA01 (losartan and diuretics)	1083	268	1.07 (0.94–1.23)	0.16494972	1.07 (0.93–1.23)	0.20272364
C09DA03 (valsartan and diuretics)	476	116	1.09 (0.88–1.33)	0.20801247	1.09 (0.89–1.34)	0.22483783
C09DA06 (candesartan and diuretics)	651	186	1.23 (1.04–1.46)	0.01576002	1.23 (1.04–1.46)	0.01862255
C09DA07 (telmisartan and diuretics)	265	69	1.14 (0.87–1.49)	0.17165784	1.14 (0.87–1.48)	0.20537980
C09DB01 (valsartan and amlodipine)	138	36	1.15 (0.79–1.66)	0.21303615	1.15 (0.79–1.66)	0.24309482
C10AA01 (simvastatin)	7647	2075	1.17 (1.11–1.24)	0.00000002	1.17 (1.11–1.23)	0.00000007
C10AA02 (lovastatin)	435	113	1.11 (0.90–1.37)	0.17090937	1.10 (0.89–1.36)	0.20537980
C10AA03 (pravastatin)	420	110	1.07 (0.87–1.33)	0.22888464	1.07 (0.86–1.32)	0.26617573
C10AA04 (fluvastatin)	652	192	1.22 (1.04–1.44)	0.01934287	1.21 (1.03–1.43)	0.02422950
C10AA05 (atorvastatin)	2079	555	1.17 (1.06–1.29)	0.00282982	1.16 (1.06–1.28)	0.00433215
C10AA07 (rosuvastatin)	653	166	1.11 (0.93–1.32)	0.14569275	1.10 (0.93–1.31)	0.17122165
C10AX09 (ezetimibe)	191	51	1.12 (0.82–1.54)	0.21463494	1.11 (0.81–1.52)	0.25508628
G03BA03 (testosterone)	74	26	1.62 (1.03–2.55)	0.03095835	1.61 (1.03–2.54)	0.03662991
G03CA03 (estradiol)	2544	626	1.13 (1.03–1.24)	0.01186695	1.13 (1.03–1.24)	0.01256433
G03FA01 (norethisterone and oestrogen)	318	69	0.98 (0.75–1.28)	0.31863949	0.99 (0.76–1.28)	0.36528071
G03FA12 (medroxyprogest‐erone and oestrogen)	107	30	1.33 (0.88–2.00)	0.11070378	1.34 (0.89–2.02)	0.11536802
H02AB06 (prednisolone)	333	158	2.00 (1.64–2.42)	0.00000000	1.95 (1.61–2.37)	0.00000000
H03AA01 (levothyroxine sodium)	4662	1224	1.13 (1.06–1.21)	0.00058159	1.13 (1.05–1.21)	0.00123771
M04AA01 (allopurinol)	517	182	1.47 (1.24–1.75)	0.00005672	1.46 (1.23–1.74)	0.00009316
M05BA04 (alendronic acid)	458	117	1.08 (0.88–1.332)	0.21239505	1.08 (0.87–1.33)	0.24735090
M05BA07 (risedronic acid)	183	49	1.03 (0.75–1.42)	0.31333937	1.02 (0.74–1.41)	0.35839871
N03AE01 (clonazepam)	207	49	1.02 (0.74–1.40)	0.31916915	0.97 (0.71–1.33)	0.35273561
N03AF01 (carbamazepine)	289	82	1.25 (0.97–1.60)	0.06508760	1.22 (0.95–1.56)	0.09537257
N03AF02 (oxcarbazepine)	116	38	1.48 (1.02–2.15)	0.03191540	1.42 (0.98–2.06)	0.05870250
N03AG01 (valproic acid)	197	62	1.38 (1.03–1.85)	0.02579197	1.31 (0.98–1.75)	0.06284107
N03AX12 (gabapentin)	95	30	1.24 (0.81–1.88)	0.16657699	1.22 (0.80–1.85)	0.20537980
N03AX16 (pregabalin)	278	60	0.93 (0.70–1.23)	0.24888674	0.91 (0.69–1.21)	0.25508628
N04BA02 (levodopa and decarboxylase inhibitor)	273	72	1.14 (0.87–1.48)	0.17090937	1.13 (0.86–1.47)	0.21325249
N04BC05 (pramipexole)	255	87	1.45 (1.13–1.86)	0.00553768	1.44 (1.12–1.85)	0.00728296
N04BD01 (selegiline)	86	21	1.07 (0.66–1.74)	0.29120875	1.06 (0.65–1.72)	0.33963447
N05AA02 (chlorpromazine)	119	29	1.08 (0.71–1.62)	0.28158437	1.01 (0.67–1.52)	0.37827752
N05AB03 (perphenazine)	159	28	0.75 (0.50–1.13)	0.11070378	0.73 (0.49–1.09)	0.09537257
N05AH03 (olanzapine)	211	38	0.79 (0.56–1.12)	0.11367182	0.74 (0.52–1.05)	0.07373444
N05AH04 (quetiapine)	247	48	0.81 (0.59–1.11)	0.11367182	0.78 (0.57–1.07)	0.09537257
N05AX08 (risperidone)	242	41	0.72 (0.51–1.00)	0.04034571	0.69 (0.49–0.96)	0.02743514
N05BA01 (diazepam)	231	63	1.20 (0.90–1.59)	0.12543592	1.16 (0.87–1.53)	0.19408861
N05BA04 (oxazepam)	712	213	1.26 (1.07–1.47)	0.00690057	1.26 (1.06–1.44)	0.01256433
N05BA06 (lorazepam)	349	62	0.80 (0.61–1.06)	0.08518865	0.79 (0.60–1.03)	0.07321678
N05BA12 (alprazolam)	329	80	1.01 (0.79–1.30)	0.32024553	0.99 (0.77–1.27)	0.37224908
N05CD07 (temazepam)	685	215	1.35 (1.15–1.57)	0.00058159	1.33 (1.14–1.56)	0.00096959
N05CF01 (zopiclone)	2305	711	1.31 (1.20–1.43)	0.00000002	1.30 (1.19–1.41)	0.00000007
N05CF02 (zolpidem)	487	129	1.13 (0.92–1.37)	0.14137117	1.12 (0.92–1.36)	0.17122165
N06AA09 (amotriptyline)	119	34	1.29 (0.88–1.90)	0.11663343	1.26 (0.86–1.85)	0.15969798
N06AA12 (doxepin)	129	35	1.11 (0.76–1.62)	0.24888674	1.10 (0.75–1.60)	0.29037235
N06AB03 (fluoxetine)	153	41	1.21 (0.85–1.71)	0.15769600	1.18 (0.83–1.66)	0.20570447
N06AB04 (citalopram)	776	194	1.07 (0.91–1.26)	0.19644968	1.05 (0.90–1.24)	0.25951214
N06AB06 (sertraline)	76	30	1.74 (1.13–2.67)	0.01521080	1.67 (1.08–2.57)	0.02422950
N06AB10 (escitalopram)	251	61	1.07 (0.81–1.43)	0.25578806	1.06 (0.80–1.41)	0.30790114
N06AX03 (mianserin)	109	25	0.98 (0.63–1.52)	0.32024553	0.97 (0.62–1.51)	0.35839871
N06AX11 (mirtazapine)	670	130	0.80 (0.66–0.97)	0.02187783	0.78 (0.65–0.95)	0.01696441
N06AX16 (venlafaxine)	149	50	1.44 (1.04–2.00)	0.02511330	1.41 (1.02–1.96)	0.03603580
N06CA01 (amitriptyline and psycoleptics)	329	97	1.26 (1.00–1.59)	0.04034571	1.24 (0.98–1.56)	0.06284107
N06DA02 (donepezil)	302	45	0.55 (0.40–0.76)	0.00072644	0.55 (0.40–0.76)	0.00094082
N06DA03 (rivastigmine)	178	17	0.35 (0.21–0.58)	0.00014541	0.35 (0.21–0.57)	0.00014306
N06DA04 (galantamine)	127	12	0.37 (0.20–0.67)	0.00210002	0.37 (0.20–0.67)	0.00232730
N06DX01 (memantine)	256	17	0.24 (0.15–0.40)	0.00000015	0.24 (0.15–0.40)	0.00000016
N07CA01 (betahistine)	317	81	1.10 (0.86–1.41)	0.21303615	1.10 (0.86–1.41)	0.23860402

*Note*: The analysis was unadjusted and adjusted for socio‐economic groups giving odds ratios (ORs) with 95% confidence intervals (CIs) with *q* values based on conditional logistic regression analyses of nested case–control design.

Results were similar after socio‐economic adjustment.

We also looked at drug classes. Results are shown in Table 3. When we considered at least two redeemed prescriptions a year in 5 years before the event, we found out that the incident cataract surgery was more strongly associated with any statin use (OR 1.20, CI 1.15–1.26). The same was true regarding AT2R antagonists as a group (OR 1.15, CI 1.07–1.24), AT2R antagonists combined with diuretics (OR 1.13, CI 1.03–1.25), ACE inhibitors (OR 1.12, CI 1.05–1.20), dihydropyridine derivatives (OR 1.19, CI 1.12–1.27), class Ic antiarrhythmics (OR 1.38, CI 1.02–1.87), organic nitrates (OR 1.49, CI 1.39–1.61), furosemide combined with potassium sparing agents (OR 1.58, CI 1.18–2.10), selective beta blockers (OR 1.22, CI 1.16–1.27), benzothiazepine derivatives (OR 1.29, CI 1.07–1.56), glucocorticoids (OR 2.00, CI 1.62–2.48), thyroid hormones (OR 1.14, CI 1.04–1.24), carboxamide derivatives (OR 1.37, CI 1.08–1.74), dopamine agonists (OR 1.42, CI 1.12–1.81), benzodiazepine derivatives (OR 1.29, CI 1.06–1.56), benzodiazepine related drugs (OR 1.19, CI 1.08–1.32) and antidepressants in combination with psycholeptics (OR 1.42, CI 1.07–1.87).

**TABLE 3:** Exposure to systemic medications by ATC groups with at least two redeemed prescriptions every year for 5 years before cataract surgery event in persons without diabetes compared to their matched controls who did not undergo surgery.

ATC group	OR.Unadj	*q*.value.Unadj	OR.Adj	*q*.value.Adj
C01AA (digitalis glycosides)	1.13 (0.89–1.43)	0.16987612	1.13 (0.89–1.43)	0.19699798
C01BC (antiarrhythmics, class Ic)	1.38 (1.02–1.87)	0.03649527	1.38 (1.02–1.88)	0.04058227
C01DA (organic nitrates)	1.49 (1.39–1.61)	0.00000000	1.48 (1.38–1.60)	0.00000000
C02AC (imidazoline receptor agonists)	1.33 (0.95–1.88)	0.07495888	1.34 (0.95–1.88)	0.08000054
C03AA (thiazides, plain)	1.15 (0.87–1.52)	0.17665767	1.14 (0.87–1.51)	0.19699798
C03BA (sulfonamides, plain)	1.20 (0.78–1.84)	0.20506548	1.21 (0.79–1.84)	0.20941263
C03CA (sulfonamides, plain)	1.33 (1.18–1.51)	0.00001991	1.32 (1.17–1.50)	0.00002992
C03DA (aldosterone antagonists)	1.16 (0.88–1.53)	0.16310469	1.15 (0.87–1.51)	0.19699798
C03EA (low‐ceiling diuretics and potassium‐sparing agents)	1.08 (0.96–1.21)	0.12257003	1.08 (0.96–1.21)	0.13752573
C03EB (high‐ceiling diuretics and potassium‐sparing agents)	1.58 (1.18–2.10)	0.00306054	1.57 (1.18–2.08)	0.00378270
C07AA (beta blocking agents, non‐selective)	1.19 (0.98–1.45)	0.06336024	1.18 (0.97–1.43)	0.08429050
C07AB (beta blocking agents, selective)	1.22 (1.16–1.27)	0.00000000	1.21 (1.16–1.27)	0.00000000
C07AG (alpha and beta blocking agents)	0.94 (0.76–1.15)	0.26250339	0.93 (0.76–1.14)	0.25057227
C07BB (beta blocking agents, selective, and thiazides)	1.02 (0.84–1.23)	0.35949704	1.01 (0.84–1.22)	0.39593700
C07FB (beta blocking agents and calcium channel blockers)	0.89 (0.71–1.13)	0.18051466	0.89 (0.71–1.13)	0.19699798
C08CA (dihydropyridine derivatives)	1.19 (1.12–1.27)	0.00000003	1.19 (1.12–1.26)	0.00000006
C08DA (phenylalkylamine derivatives)	0.72 (0.49–1.05)	0.06766939	0.71 (0.49–1.05)	0.07087684
C08DB (benzothiazepine derivatives)	1.29 (1.07–1.56)	0.00928002	1.28 (1.07–1.55)	0.01234126
C09AA (ACE inhibitors, plain)	1.12 (1.05–1.20)	0.00181067	1.11 (1.04–1.19)	0.00370059
C09BA (ACE inhibitors and diuretics)	1.09 (0.96–1.23)	0.12257003	1.08 (0.96–1.22)	0.13935375
C09CA (angiotensin II receptor blockers (ARBs), plain)	1.15 (1.07–1.24)	0.00026133	1.15 (1.07–1.23)	0.00031353
C09DA (angiotensin II receptor blockers (ARBs) and diuretics)	1.13 (1.03–1.25)	0.01213105	1.13 (1.03–1.24)	0.01594015
C09DB (angiotensin II receptor blockers (ARBs) and calcium channel blockers)	1.27 (0.85–1.89)	0.13941741	1.26 (0.85–1.88)	0.16076319
C10AA (HMG CoA reductase inhibitors)	1.20 (1.15–1.26)	0.00000000	1.17 (1.14–1.25)	0.00000000
C10AX (other lipid modifying agents)	1.26 (0.89–1.78)	0.12432364	1.24 (0.88–1.75)	0.15026580
G03CA (natural and semisynthetic oestrogens, plain)	1.01 (0.89–1.14)	0.37058978	1.01 (0.90–1.14)	0.38717469
G03FA (Progestogens and oestrogens, fixed combinations)	0.93 (0.72–1.20)	0.26693618	0.94 (0.73–1.21)	0.30239001
G03FB (progestogens and oestrogens, sequential preparations)	1.06 (0.64–1.74)	0.35949704	1.03 (0.62–1.70)	0.39593700
H02AB (glucocorticoids)	2.01 (1.62–2.48)	0.00000000	1.97 (1.59–2.43)	0.00000000
H03AA (thyroid hormones)	1.14 (1.04–1.24)	0.00615105	1.13 (1.04–1.23)	0.00912288
M04AA (preparations inhibiting uric acid production)	1.65 (1.32–2.06)	0.00002185	1.64 (1.32–2.05)	0.00002992
M05BA (bisphosphonates)	1.12 (0.93–1.34)	0.13759712	1.11 (0.93–1.33)	0.16076319
N03AE (benzodiazepine derivatives)	0.86 (0.55–1.37)	0.26250339	0.82 (0.52–1.30)	0.20941263
N03AF (carboxamide derivatives)	1.37 (1.08–1.74)	0.01201102	1.33 (1.05–1.68)	0.02398878
N03AG (fatty acid derivatives)	1.31 (0.95–1.82)	0.07495888	1.24 (0.90–1.73)	0.13752573
N03AX (other antiepileptics)	1.13 (0.92–1.39)	0.13941741	1.10 (0.89–1.35)	0.20728787
N04BA (dopa and dopa derivatives)	1.25 (0.99–1.59)	0.05727483	1.24 (0.98–1.57)	0.07009968
N04BC (dopamine agonists)	1.42 (1.12–1.81)	0.00615105	1.40 (1.10–1.79)	0.00912288
N05AA (phenothiazines with aliphatic side‐chain)	1.13 (0.73–1.77)	0.26693618	1.06 (0.68–1.65)	0.38587058
N05AB (phenothiazines with piperazine structure)	0.76 (0.48–1.18)	0.13759712	0.73 (0.46–1.14)	0.12477867
N05AF (thioxanthene derivatives)	1.02 (0.62–1.65)	0.38683518	0.95 (0.57–1.55)	0.38717469
N05AH (diazepines, oxazepines, thiazepines and oxepines)	0.80 (0.63–1.02)	0.06336024	0.76 (0.59–0.97)	0.02721651
N05AX (other antipsychotics)	0.74 (0.51–1.08)	0.08528605	0.70 (0.48–1.03)	0.06314057
N05BA (benzodiazepine derivatives)	1.01 (0.89–1.16)	0.35949704	0.99 (0.87–1.13)	0.38717469
N05CD (benzodiazepine derivatives)	1.29 (1.06–1.56)	0.01213105	1.27 (1.05–1.54)	0.01777174
N05CF (benzodiazepine related drugs)	1.19 (1.08–1.32)	0.00146967	1.18 (1.07–1.31)	0.00316714
N06AA (non‐selective monoamine reuptake inhibitors)	1.34 (1.02–1.77)	0.03649527	1.32 (1.00–1.74)	0.05048063
N06AB (selective serotonin reuptake inhibitors)	1.16 (1.01–1.34)	0.03546391	1.14 (0.99–1.31)	0.06314057
N06AX (other antidepressants)	0.98 (0.83–1.15)	0.34262190	0.96 (0.81–1.13)	0.29597145
N06CA (antidepressants in combination with psycholeptics)	1.42 (1.07–1.87)	0.01583302	1.39 (1.05–1.84)	0.02398878
N06DA (anticholinesterases)	0.50 (0.39–0.64)	0.00000013	0.50 (0.39–0.64)	0.00000014
N06DX (other anti‐dementia drugs)	0.30 (0.18–0.50)	0.00001198	0.30 (0.18–0.50)	0.00001227
N07CA (antivertigo preparations)	0.92 (0.67–1.25)	0.26693618	0.91 (0.67–1.25)	0.29349417

*Note*: The analysis was unadjusted and adjusted for socio‐economic groups giving odds ratios (ORs) with 95% confidence intervals (CIs) with *q* values based on conditional logistic regression analyses of nested case–control design.

Results were similar after socio‐economic adjustment.

### 3.3 Systemic neuropsychiatric drug use in the study population associated with reduced risk of surgically treated cataract

Both after no adjustment and/or socio‐economic adjustment, our analysis revealed that the use of certain neuropsychiatric drugs was negatively associated with cataract surgery. These included the use of memantine (OR 0.24; 0.15–0.40), risperidone (0.72; 0.51–1.00), rivastigmine (0.35; 0.21–0.58) and donepezil (0.55; 0.40–0.76).

When considered as a group, both anticholinesterases (OR 0.50, CI 0.39–0.64) and other anti‐dementia drugs (OR 0.30, CI 0.18–0.50) were negatively associated with cataract surgery.

### 3.4 Exposure definition 2, trend

Trend was also calculated as an alternative way to define exposure to systemic drugs. Results are shown in Table 4.

**TABLE 4:** Exposure to systemic medications for a prescription (linear trend for number of prescriptions) in 5 years before CJE20 event in persons without diabetes who underwent cataract surgery compared to their matched controls who did not undergo surgery.

ATC	OR.Unadj	q.value.Unadj	OR.Adj	q.value.Adj
A08AA10 (sibutramine)	0.75 (0.13–4.40)	0.84789139	0.79 (0.14–4.50)	0.93524243
A08AB01 (orlistat)	1.26 (0.79–2.00)	0.56007640	1.27 (0.80–2.02)	0.59352994
C01AA05 (digoxin)	1.05 (1.03–1.08)	0.00045366	1.05 (1.03–1.08)	0.00074049
C01BA01 (quinidine)	1.04 (0.88–1.23)	0.83091313	1.04 (0.87–1.23)	0.92438759
C01BA03 (disopyramide)	0.98 (0.84–1.14)	0.86792972	0.98 (0.84–1.14)	0.93640521
C01BB02 (mexiletine)	1.19 (0.68–2.08)	0.74650787	1.19 (0.68–2.08)	0.81962616
C01BC03 (propafenone)	1.11 (0.90–1.37)	0.54718607	1.11 (0.90–1.37)	0.59352994
C01BC04 (flecainide)	1.07 (1.02–1.13)	0.02308976	1.07 (1.02–1.13)	0.02876574
C01BD01 (amiodarone)	1.01 (0.91–1.12)	0.87115713	1.01 (0.91–1.12)	0.93873173
C01BD07 (dronedarone)	1.07 (0.85–1.35)	0.76487335	1.06 (0.85–1.34)	0.86480247
C01CA01 (etilefrine)	1.02 (0.77–1.35)	0.90623117	1.01 (0.77–1.34)	0.98690232
C01CA24 (epinephrine)	1.27 (0.84–1.93)	0.47752275	1.24 (0.82–1.89)	0.59075294
C01DA02 (glyceryl trinitrate)	1.10 (1.07–1.14)	0.00000000	1.10 (1.07–1.13)	0.00000000
C01DA08 (isosorbide dinitrate)	1.09 (1.06–1.11)	0.00000000	1.09 (1.06–1.11)	0.00000000
C01DA14 (isosorbide mononitrate)	1.10 (1.09–1.12)	0.00000000	1.10 (1.09–1.12)	0.00000000
C01DA70 (organic nitrates in combination with psycholeptics)	1.24 (0.71–2.16)	0.68870605	1.24 (0.71–2.16)	0.75502444
C02AC01 (clonidine)	0.91 (0.56–1.48)	0.84017664	0.91 (0.56–1.48)	0.92995513
C02AC05 (moxonidine)	1.07 (1.01–1.12)	0.05857119	1.07 (1.01–1.12)	0.06555219
C02CA01 (prazosin)	1.01 (0.91–1.12)	0.88568201	1.01 (0.91–1.12)	0.97566108
C02KX01 (bosentan)	1.09 (0.60–1.95)	0.85229795	1.09 (0.61–1.97)	0.92995513
C03AA03 (hydrochlorothiazide)	1.03 (0.99–1.06)	0.20079331	1.02 (0.99–1.06)	0.24750319
C03BA11 (indapamide)	1.07 (0.99–1.14)	0.15050556	1.07 (1.00–1.14)	0.16725247
C03CA01 (furosemide)	1.07 (1.05–1.08)	0.00000000	1.06 (1.05–1.08)	0.00000000
C03DA01 (spironolactone)	1.07 (1.03–1.11)	0.00105912	1.07 (1.03–1.11)	0.00205394
C03DB01 (amiloride)	0.68 (0.17–2.69)	0.76608321	0.68 (0.17–2.68)	0.85462416
C03EA01 (hydrochlorothiazide and potassium‐sparing agents)	1.01 (0.99–1.03)	0.44656652	1.01 (0.99–1.03)	0.50589985
C03EA02 (trichlormethiazide and potassium‐sparing agents)	1.07 (1.02–1.12)	0.03278177	1.07 (1.02–1.12)	0.03560429
C03EB01 (furosemide and potassium‐sparing agents)	1.06 (1.02–1.10)	0.02250977	1.06 (1.02–1.10)	0.03313976
C04AE01 (ergoloid mesylates)	0.98 (0.87–1.11)	0.84789139	0.98 (0.87–1.11)	0.92995513
C04AX01 (cyclandelate)	1.74 (1.00–3.03)	0.14624678	1.74 (0.99–3.02)	0.16725247
C05AA08 (fluocortolone)	1.06 (0.96–1.17)	0.45122237	1.06 (0.96–1.17)	0.52263353
C07AA03 (pindolol)	1.08 (0.94–1.08)	0.87804888	1.01 (0.94–1.08)	0.95010283
C07AA05 (propranolol)	1.06 (1.02–1.10)	0.01641902	1.06 (1.02–1.10)	0.03111892
C07AA06 (timolol)	0.96 (0.76–1.22)	0.84789139	0.96 (0.75–1.21)	0.92995513
C07AA07 (sotalol)	1.04 (0.99–1.09)	0.19208189	1.04 (0.99–1.09)	0.24464831
C07AB02 (metoprolol)	1.02 (1.01–1.03)	0.00333298	1.02 (1.01–1.03)	0.00574637
C07AB03 (atenolol)	1.05 (1.02–1.080)	0.00333298	1.05 (1.02–1.08)	0.00450522
C07AB04 (acebutolol)	1.05 (1.00–1.11)	0.14624678	1.05 (0.99–1.11)	0.18100206
C07AB05 (betaxolol)	1.01 (0.97–1.06)	0.83716663	1.01 (0.96–1.06)	0.92995513
C07AB07 (bisoprolol)	1.05 (1.04–1.06)	0.00000000	1.05 (1.04–1.06)	0.00000000
C07AB08 (celiprolol)	1.07 (1.02–1.11)	0.02244824	1.06 (1.02–1.11)	0.03246326
C07AB12 (nebivolol)	0.97 (0.85–1.10)	0.76711330	0.96 (0.85–1.09)	0.82877978
C07AG01 (labetalol)	0.96 (0.52–1.77)	0.90039400	0.96 (0.52–1.77)	0.98491241
C07AG02 (carvedilol)	1.01 (0.98–1.04)	0.64855267	1.01 (0.98–1.04)	0.75817688
C07BB02 (metoprolol and thiazides)	1.03 (0.97–1.09)	0.56454288	1.03 (0.97–1.09)	0.64604922
C07BB07 (bisoprolol and thiazides)	1.01 (0.97–1.04)	0.83091313	1.01 (0.97–1.04)	0.92995513
C07BB12 (nebivolol and thiazides)	1.72 (0.47–6.36)	0.64855267	1.70 (0.46–6.28)	0.72646082
C07FB02 (metoprolol and felodipine)	0.99 (0.95–1.03)	0.84017664	0.99 (0.95–1.03)	0.92995513
C07FB03 (atenolol and nifedipine)	1.08 (0.84–1.38)	0.74650787	1.08 (0.85–1.39)	0.80389855
C08CA01 (amlodipine)	1.04 (1.02–1.05)	0.00000976	1.03 (1.02–1.05)	0.00001982
C08CA02 (felodipine)	1.04 (1.02–1.06)	0.00165667	1.04 (1.02–1.06)	0.00218145
C08CA03 (isradipine)	0.99 (0.91–1.07)	0.84789139	0.99 (0.91–1.07)	0.92995513
C08CA05 (nifedipine)	1.05 (1.02–1.08)	0.02244824	1.05 (1.01–1.08)	0.03153513
C08CA07 (nisoldipine)	1.05 (0.97–1.14)	0.48152578	1.05 (0.97–1.14)	0.54980764
C08CA10 (nilvadipine)	1.05 (0.99–1.10)	0.25060892	1.04 (0.99–1.10)	0.30295114
C08CA13 (lercanidipine)	1.04 (1.01–1.06)	0.01587700	1.03 (1.01–1.06)	0.02222815
C08DA01 (verapamil)	0.99 (0.95–1.05)	0.90623117	0.99 (0.95–1.05)	0.98690232
C08DB01 (diltiazem)	1.05 (1.02–1.08)	0.00808080	1.05 (1.02–1.08)	0.01107405
C09AA01 (captopril)	1.07 (1.01–1.14)	0.09860145	1.07 (1.01–1.13)	0.12382731
C09AA02 (enalapril)	1.02 (1.01–1.04)	0.02308976	1.02 (1.01–1.04)	0.04471550
C09AA03 (lisinopril)	1.01 (0.97–1.05)	0.75487249	1.01 (0.97–1.05)	0.82368409
C09AA04 (perindopril)	1.06 (1.02–1.10)	0.02174879	1.06 (1.02–1.10)	0.02327554
C09AA05 (ramipril)	1.04 (1.02–1.05)	0.00008129	1.04 (1.02–1.05)	0.00015545
C09AA06 (quinapril)	0.87 (0.74–1.02)	0.22077148	0.87 (0.74–1.02)	0.24750319
C09BA02 (enalapril and diuretics)	1.00 (0.98–1.02)	0.91631635	0.99 (0.98–1.02)	0.98690232
C09BA03 (lisinopril and diuretics)	1.00 (0.95–1.05)	0.91631635	1.00 (0.95–1.05)	0.99718992
C09BA04 (perindopril and diuretics)	1.01 (0.94–1.10)	0.84789139	1.01 (0.94–1.10)	0.92995513
C09BA05 (ramipril and diuretics)	1.01 (0.94–1.09)	0.85337459	1.01 (0.94–1.09)	0.93873173
C09BA06 (quinapril and diuretics)	0.98 (0.82–1.17)	0.85229795	0.97 (0.82–1.16)	0.92995513
C09BB02 (enalapril and lercanidipine)	0.99 (0.82–1.21)	0.90623117	0.99 (0.82–1.21)	0.98690232
C09BB04 (perindopril and amlodipine)	1.01 (0.88–1.15)	0.90623117	1.01 (0.88–1.16)	0.98111579
C09BB05 (ramipril and felodipine)	1.01 (0.90–1.15)	0.87022015	1.02 (0.90–1.15)	0.92995513
C09BX01 (perindopril, amlodipine and indapamide)	1.13 (0.36–3.58)	0.87297634	1.13 (0.36–3.59)	0.95010283
C09CA (angiotensin II receptor blockers (ARBs), plain)	1.04 (1.02–1.05)	0.00000009	1.04 (1.02–1.05)	0.00000013
C09CA01 (losartan)	1.05 (1.03–1.07)	0.00001205	1.05 (1.03–1.07)	0.00001982
C09CA02 (eprosartan)	0.96 (0.89–1.05)	0.63173511	0.96 (0.89–1.05)	0.67535477
C09CA03 (valsartan)	1.02 (0.99–1.06)	0.31910857	1.02 (0.99–1.06)	0.35940872
C09CA06 (candesartan)	1.03 (1.01–1.05)	0.03513752	1.03 (1.01–1.05)	0.03984701
C09CA07 (telmisartan)	1.04 (0.99–1.08)	0.20676290	1.04 (0.99–1.08)	0.22528756
C09CA08 (olmesartan medoxomil)	1.03 (0.95–1.11)	0.71279746	1.03 (0.95–1.12)	0.75426915
C09DA01 (losartan and diuretics)	1.02 (1.00–1.05)	0.14624678	1.02 (1.00–1.05)	0.17170111
C09DA02 (eprosartan and diuretics)	1.02 (0.91–1.14)	0.84497283	1.02 (0.91–1.14)	0.92995513
C09DA03 (valsartan and diuretics)	1.02 (0.99–1.06)	0.42440275	1.02 (0.99–1.06)	0.44407262
C09DA06 (candesartan and diuretics)	1.05 (1.02–1.08)	0.00444053	1.05 (1.02–1.08)	0.00450522
C09DA07 (telmisartan and diuretics)	1.05 (1.01–1.10)	0.09124976	1.05 (1.01–1.10)	0.09534366
C09DA08 (olmesartan medoxomil and diuretics)	1.05 (0.89–1.23)	0.75978173	1.05 (0.89–1.23)	0.86306239
C09DB01 (valsartan and amlodipine)	1.03 (0.97–1.10)	0.52673946	1.03 (0.97–1.10)	0.57057018
C09DB02 (olmesartan medoxomil and amlodipine)	0.58 (0.28–1.21)	0.31945491	0.59 (0.29–1.23)	0.37382090
C09DX01 (valsartan, amlodipine and hydrochlorothiazide)	1.02 (0.90–1.15)	0.85229795	1.01 (0.90–1.14)	0.93924797
C09DX04 (valsartan and sacubitril)	0.00 (0.00–92 639)	0.90623117	0.00 (0.00–49 528)	0.98690232
C10AA01 (simvastatin)	1.04 (1.03–1.05)	0.00000000	1.04 (1.03–1.05)	0.00000000
C10AA02 (lovastatin)	1.03 (0.99–1.07)	0.34364038	1.03 (0.99–1.06)	0.40572489
C10AA03 (pravastatin)	1.04 (1.00–1.07)	0.10592792	1.04 (1.00–1.07)	0.14063588
C10AA04 (fluvastatin)	1.05 (1.02–1.08)	0.00333298	1.05 (1.02–1.08)	0.00450522
C10AA05 (atorvastatin)	1.03 (1.02–1.05)	0.00040139	1.03 (1.02–1.05)	0.00059354
C10AA06 (cerivastatin)	0.00 (0.00–76 514)	0.90623117	0.00 (0.00–71 708)	0.98690232
C10AA07 (rosuvastatin)	1.04 (1.01–1.07)	0.03459285	1.04 (1.01–1.07)	0.04413624
C10AB02 (bezafibrate)	1.14 (0.92–1.41)	0.46561744	1.13 (0.91–1.40)	0.54980764
C10AB04 (gemfibrozil)	1.01 (0.77–1.34)	0.90623117	1.02 (0.77–1.35)	0.98491241
C10AB05 (fenofibrate)	1.02 (0.89–1.17)	0.86216137	1.02 (0.89–1.17)	0.93873173
C10AC01 (colestyramine)	1.04 (0.83–1.31)	0.84017664	1.04 (0.83–1.31)	0.92995513
C10AC02 (colestipol)	1.23 (0.82–1.83)	0.52673946	1.22 (0.82–1.82)	0.61229025
C10AX09 (ezetimibe)	1.02 (0.97–1.07)	0.74650787	1.02 (0.96–1.07)	0.85462416
C10BA02 (simvastatin and ezetimibe)	1.06 (0.80–1.41)	0.83716663	1.06 (0.80–1.41)	0.92189224
G03BA03 (testosterone)	1.12 (1.04–1.21)	0.01763862	1.12 (1.04–1.20)	0.02222815
G03BB01 (mesterolone)	0.94 (0.44–1.99)	0.89264812	0.94 (0.44–1.99)	0.97566108
G03CA03 (estradiol)	1.04 (1.02–1.05)	0.00011717	1.04 (1.02–1.05)	0.00010759
G03CA04 (estriol)	1.00 (0.90–1.10)	0.90623117	0.99 (0.90–1.10)	0.98690232
G03CA57 (conjugated oestrogens)	0.00 (0.00–40 669)	0.90623117	0.00 (0.00–40 669)	0.98690232
G03CC04 (estrone)	0.97 (0.48–1.94)	0.90623117	0.96 (0.48–1.94)	0.98690232
G03CX01 (tibolone)	1.17 (1.08–1.26)	0.00116477	1.17 (1.08–1.26)	0.00099141
G03DA02 (medroxyprogesterone)	1.01 (0.84–1.21)	0.90623117	1.01 (0.84–1.21)	0.98690232
G03DB01 (dydrogesterone)	1.06 (0.88–1.27)	0.74650787	1.06 (0.89–1.28)	0.79540024
G03DB02 (megestrol)	0.70 (0.23–2.11)	0.73923610	0.70 (0.23–2.12)	0.81163728
G03DC02 (norethisterone)	0.28 (0.05–1.61)	0.32370004	0.27 (0.05–1.61)	0.35940872
G03DC03 (lynestrenol)	1.73 (0.90–3.31)	0.23835842	1.73 (0.90–3.31)	0.25698079
G03DC05 (G03DC05)	1.06 (0.95–1.18)	0.51022772	1.06 (0.95–1.19)	0.54980764
G03FA01 (norethisterone and oestrogen)	1.01 (0.97–1.05)	0.87022015	1.01 (0.97–1.05)	0.92995513
G03FA12 (medroxyprogesterone and oestrogen)	1.04 (0.98–1.11)	0.43989401	1.04 (0.98–1.11)	0.43880679
G03FA14 (dydrogesterone and oestrogen)	0.94 (0.84–1.06)	0.52673946	0.95 (0.85–1.06)	0.61871275
G03FA17 (drospirenone and oestrogen)	1.07 (0.91–1.25)	0.64855267	1.07 (0.91–1.25)	0.70742367
G03FB05 (norethisterone and oestrogen)	0.98 (0.88–1.09)	0.83091313	0.97 (0.88–1.08)	0.87347840
G03FB06 (medroxyprogesterone and oestrogen)	1.09 (0.99–1.20)	0.20792164	1.09 (0.99–1.20)	0.25138403
G03FB08 (dydrogesterone and oestrogen)	1.13 (0.91–1.40)	0.48152578	1.13 (0.91–1.41)	0.53136610
G03FB09 (levonorgestrel and oestrogen)	1.00 (0.74–1.35)	0.91631635	1.00 (0.74–1.35)	0.99718992
G03FB11 (trimegestone and oestrogen)	1.28 (0.13–12.8)	0.87297634	1.30 (0.13–13.0)	0.94916472
G03HA01 (cyproterone)	1.11 (0.80–1.54)	0.74650787	1.11 (0.80–1.54)	0.81962616
G03HB01 (cyproterone and oestrogen)	1.51 (0.80–2.85)	0.41121956	1.54 (0.81–2.90)	0.42740107
G03XC01 (raloxifene)	1.07 (0.90–1.27)	0.69081147	1.07 (0.90–1.27)	0.76670259
H01BA02 (desmopressin)	1.11 (0.95–1.30)	0.38200223	1.10 (0.95–1.29)	0.46115372
H01CB02 (octreotide)	0.94 (0.66–1.32)	0.84017664	0.93 (0.66–1.31)	0.92800761
H01CB03 (lanreotide)	0.00 (0.00–21 323)	0.90623117	0.00 (0.00–40 456)	0.98690232
H02AA02 (fludrocortisone)	0.91 (0.73–1.14)	0.64774570	0.91 (0.73–1.13)	0.68457110
H02AB02 (dexamethasone)	1.68 (1.35–2.09)	0.00002876	1.69 (1.36–2.10)	0.00002646
H02AB04 (methylprednisolone)	1.23 (1.15–1.32)	0.00000001	1.23 (1.15–1.31)	0.00000004
H02AB06 (prednisolone)	1.22 (1.19–1.25)	0.00000000	1.21 (1.18–1.24)	0.00000000
H02AB07 (prednisone)	1.17 (1.10–1.24)	0.00000415	1.16 (1.10–1.24)	0.00000782
H02AB09 (hydrocortisone)	1.04 (0.93–1.15)	0.73118168	1.04 (0.93–1.15)	0.79540024
H03AA01 (levothyroxine sodium)	1.03 (1.02–1.04)	0.00014655	1.03 (1.01–1.04)	0.00039542
H03BB01 (carbimazole)	1.05 (0.90–1.22)	0.76466766	1.04 (0.89–1.22)	0.87347840
M04AA01 (allopurinol)	1.09 (1.06–1.12)	0.00000000	1.09 (1.06–1.12)	0.00000000
M04AA03 (febuxostat)	1.54 (0.87–2.71)	0.30652279	1.53 (0.87–2.70)	0.34207047
M04AB01 (probenecid)	0.82 (0.22–3.03)	0.84789139	0.82 (0.22–3.02)	0.92995513
M05BA01 (etidronic acid)	1.02 (0.88–1.19)	0.84789139	1.02 (0.88–1.19)	0.93873173
M05BA02 (clodronic acid)	1.11 (0.91–1.35)	0.51764789	1.11 (0.91–1.34)	0.59352994
M05BA04 (alendronic acid)	1.06 (1.03–1.09)	0.00036695	1.06 (1.03–1.09)	0.00072421
M05BA06 (ibandronic acid)	1.09 (1.00–1.18)	0.12384922	1.08 (1.00–1.17)	0.16725247
M05BA07 (risedronic acid)	1.06 (1.01–1.10)	0.03602078	1.06 (1.01–1.10)	0.05054109
M05BA08 (zoledronic acid)	0.00 (0.00–11 556)	0.90623117	0.00 (0.00–26 989)	0.98690232
M05BB03 (alendronic acid and colecalciferol)	1.05 (0.99–1.11)	0.17213731	1.05 (0.99–1.11)	0.22908542
M05BB04 (risedronic acid, calcium and colecalciferol, sequential)	1.06 (0.71–1.59)	0.84789139	1.07 (0.72–1.59)	0.92995513
M05BX03 (strontium ranelate)	1.06 (0.88–1.29)	0.73923610	1.06 (0.88–1.29)	0.80389855
M05BX04 (denosumab)	1.13 (0.98–1.29)	0.23656381	1.12 (0.98–1.29)	0.26569749
N03AA03 (primidone)	1.36 (0.81–2.27)	0.46334916	1.36 (0.81–2.27)	0.51726312
N03AB02 (phenytoin)	0.97 (0.88–1.07)	0.73923610	0.96 (0.88–1.06)	0.75817688
N03AE01 (clonazepam)	1.02 (0.97–1.07)	0.71531554	1.01 (0.96–1.06)	0.92995513
N03AF01 (carbamazepine)	1.06 (1.01–1.10)	0.06158765	1.05 (1.01–1.10)	0.11054164
N03AF02 (oxcarbazepine)	1.09 (1.02–1.16)	0.03852154	1.08 (1.01–1.16)	0.07201014
N03AG01 (valproic acid)	1.03 (0.98–1.08)	0.52449079	1.02 (0.97–1.07)	0.79540024
N03AG06 (tiagabine)	1.51 (0.91–2.51)	0.25060892	1.51 (0.91–2.51)	0.27835027
N03AX09 (lamotrigine)	1.11 (1.02–1.21)	0.04908703	1.11 (1.02–1.20)	0.08565458
N03AX11 (topiramate)	1.20 (0.99–1.46)	0.17514402	1.18 (0.97–1.43)	0.25511153
N03AX12 (gabapentin)	1.10 (1.03–1.17)	0.02244824	1.09 (1.02–1.16)	0.03844897
N03AX14 (levetiracetam)	1.03 (0.93–1.14)	0.76466766	1.02 (0.92–1.13)	0.92995513
N03AX15 (zonisamide)	1.07 (0.70–1.65)	0.84789139	1.06 (0.69–1.64)	0.93524243
N03AX16 (pregabalin)	1.05 (1.01–1.09)	0.03194923	1.05 (1.01–1.09)	0.05906890
N03AX18 (lacosamide)	3.40 (1.22–9.47)	0.06817822	3.32 (1.19–9.21)	0.08540182
N04AA02 (biperiden)	0.88 (0.79–0.99)	0.08525570	0.87 (0.78–0.97)	0.05779266
N04BA02 (levodopa and decarboxylase inhibitor)	1.04 (0.99–1.08)	0.24954214	1.03 (0.99–1.08)	0.31804452
N04BA03 (levodopa, decarboxylase inhibitor and COMT inhibitor)	1.01 (0.92–1.10)	0.88550832	1.00 (0.92–1.10)	0.98690232
N04BB01 (amantadine)	0.75 (0.48–1.18)	0.42984919	0.74 (0.47–1.17)	0.44407262
N04BC01 (bromocriptine)	5.49 (0.97–31.04)	0.15269534	5.48 (0.97–31.10)	0.17230555
N04BC02 (pergolide)	1.10 (0.75–1.61)	0.81367044	1.11 (0.75–1.62)	0.86833480
N04BC04 (ropinirole)	0.98 (0.88–1.09)	0.84789139	0.98 (0.88–1.09)	0.92189224
N04BC05 (pramipexole)	1.10 (1.06–1.15)	0.00001419	1.10 (1.06–1.15)	0.00002690
N04BC06 (cabergoline)	1.01 (0.78–1.31)	0.90623117	1.02 (0.78–1.32)	0.98690232
N04BC09 (rotigotine)	1.32 (0.84–2.08)	0.45409435	1.27 (0.81–2.00)	0.58573845
N04BD01 (selegiline)	1.04 (0.97–1.12)	0.51330722	1.04 (0.97–1.12)	0.59075294
N04BD02 (rasagiline)	1.14 (0.99–1.30)	0.15822634	1.13 (0.99–1.29)	0.21176881
N04BX02 (entacapone)	1.16 (1.01–1.33)	0.11380186	1.15 (1.00–1.32)	0.15742211
N05AA01 (chlorpromazine)	1.06 (0.91–1.25)	0.69081147	1.05 (0.89–1.23)	0.86306239
N05AA02 (levomepromazine)	1.01 (0.95–1.08)	0.84017664	1.00 (0.93–1.07)	0.99718992
N05AB01 (dixyrazine)	1.78 (1.10–2.89)	0.06817822	1.77 (1.08–2.89)	0.08696639
N05AB02 (fluphenazine)	0.83 (0.51–1.34)	0.67091918	0.81 (0.50–1.32)	0.68957232
N05AB03 (perphenazine)	0.91 (0.85–0.99)	0.06817822	0.91 (0.84–0.98)	0.05022339
N05AB04 (prochlorperazine)	0.93 (0.74–1.16)	0.73657933	0.93 (0.74–1.16)	0.79540024
N05AC01 (periciazine)	0.75 (0.54–1.04)	0.20932285	0.75 (0.54–1.03)	0.22528756
N05AC02 (thioridazine)	0.69 (0.51–0.94)	0.06446136	0.68 (0.50–0.93)	0.06178545
N05AD01 (haloperidol)	0.92 (0.82–1.03)	0.30652279	0.91 (0.81–1.02)	0.25138403
N05AD03 (melperone)	0.89 (0.76–1.05)	0.37282661	0.89 (0.75–1.05)	0.37455975
N05AE03 (sertindole)	0.00 (0.00–12 033)	0.90623117	0.00 (0.00–40 913)	0.98690232
N05AE04 (ziprasidone)	0.00 (0.00–16 692)	0.90623117	0.00 (0.00–36 723)	0.98690232
N05AF01 (flupentixol)	1.09 (0.93–1.28)	0.52673946	1.08 (0.92–1.27)	0.63802424
N05AF03 (chlorprothixene)	1.06 (0.97–1.16)	0.37282661	1.05 (0.96–1.15)	0.58573845
N05AF05 (zuclopenthixol)	0.88 (0.74–1.03)	0.24948248	0.86 (0.73–1.02)	0.22528756
N05AH02 (clozapine)	0.96 (0.82–1.13)	0.79793759	0.93 (0.80–1.09)	0.67535477
N05AH03 (olanzapine)	0.92 (0.86–0.98)	0.03194923	0.91 (0.85–0.97)	0.01378089
N05AH04 (quetiapine)	0.92 (0.88–0.97)	0.00444053	0.91 (0.87–0.96)	0.00201896
N05AL01 (sulpiride)	0.93 (0.80–1.09)	0.62562988	0.93 (0.79–1.08)	0.61229025
N05AN01 (lithium)	1.10 (1.00–1.21)	0.13567492	1.09 (0.99–1.19)	0.23930221
N05AX08 (risperidone)	0.80 (0.75–0.84)	0.00000000	0.79 (0.75–0.84)	0.00000000
N05AX12 (aripiprazole)	0.71 (0.44–1.14)	0.32370004	0.70 (0.43–1.12)	0.32985370
N05AX13 (paliperidone)	0.00 (0.00–16 678)	0.90623117	0.00 (0.00–15 927)	0.98690232
N05BA01 (diazepam)	1.04 (0.99–1.08)	0.24948248	1.03 (0.99–1.07)	0.43571596
N05BA02 (chlordiazepoxide)	1.03 (0.91–1.16)	0.83091313	1.02 (0.90–1.15)	0.92995513
N05BA04 (oxazepam)	1.03 (1.00–1.06)	0.08498817	1.03 (1.00–1.05)	0.16725247
N05BA06 (lorazepam)	0.93 (0.88–0.97)	0.01106050	0.92 (0.88–0.97)	0.00678426
N05BA09 (clobazam)	0.92 (0.70–1.20)	0.73923610	0.91 (0.70–1.19)	0.79540024
N05BA12 (alprazolam)	1.02 (0.98–1.06)	0.66448062	1.01 (0.97–1.06)	0.84430418
N05BB01 (hydroxyzine)	1.06 (0.39–2.90)	0.90623117	1.06 (0.39–2.89)	0.98690232
N05BB51 (hydroxyzine, combinations)	1.64 (0.43–6.28)	0.70395948	1.64 (0.43–6.28)	0.76670259
N05BE01 (buspirone)	1.03 (0.88–1.22)	0.84017664	1.03 (0.87–1.21)	0.92995513
N05CB02 (barbiturates in combination with other drugs)	0.00 (0.00–78 807)	0.90623117	0.00 (0.00–78 807)	0.98690232
N05CD02 (nitrazepam)	1.05 (0.96–1.14)	0.50518980	1.05 (0.96–1.14)	0.57057018
N05CD05 (triazolam)	1.22 (0.86–1.71)	0.48152578	1.21 (0.86–1.71)	0.54980764
N05CD07 (temazepam)	1.04 (1.01–1.07)	0.02244824	1.04 (1.01–1.06)	0.03837322
N05CD08 (midazolam)	0.70 (0.37–1.34)	0.50859936	0.71 (0.37–1.34)	0.57057018
N05CF01 (zopiclone)	1.06 (1.05–1.08)	0.00000000	1.06 (1.05–1.08)	0.00000000
N05CF02 (zolpidem)	1.04 (1.01–1.07)	0.04908703	1.04 (1.01–1.07)	0.06043971
N05CM02 (clomethiazole)	0.80 (0.18–3.63)	0.84789139	0.80 (0.18–3.63)	0.92995513
N06AA04 (clomipramine)	1.09 (0.94–1.25)	0.46772300	1.08 (0.94–1.25)	0.54980764
N06AA06 (trimipramine)	1.02 (0.90–1.17)	0.84789139	1.02 (0.89–1.17)	0.93524243
N06AA09 (amitriptyline)	1.07 (1.01–1.13)	0.06817822	1.06 (1.00–1.12)	0.12382731
N06AA10 (nortriptyline)	0.88 (0.68–1.16)	0.60268141	0.88 (0.67–1.16)	0.64920820
N06AA12 (doxepin)	1.02 (0.96–1.09)	0.74650787	1.02 (0.95–1.08)	0.87229348
N06AB03 (fluoxetine)	1.04 (0.99–1.11)	0.31945491	1.04 (0.98–1.10)	0.45245994
N06AB04 (citalopram)	1.00 (0.98–1.03)	0.90623117	0.99 (0.97–1.02)	0.97566108
N06AB05 (paroxetine)	1.13 (1.04–1.22)	0.02308976	1.12 (1.03–1.22)	0.03836837
N06AB06 (sertraline)	1.08 (1.01–1.16)	0.06817822	1.08 (1.01–1.15)	0.11671514
N06AB08 (fluvoxamine)	1.02 (0.84–1.23)	0.89264812	1.01 (0.84–1.22)	0.98491241
N06AB10 (escitalopram)	1.01 (0.97–1.05)	0.83091313	1.01 (0.97–1.05)	0.92995513
N06AG02 (moclobemide)	1.15 (1.00–1.31)	0.14368647	1.14 (0.99–1.31)	0.17170111
N06AX03 (mianserin)	0.99 (0.92–1.06)	0.83716663	0.98 (0.91–1.06)	0.87347840
N06AX05 (trazodone)	1.11 (0.956–1.29)	0.36066276	1.10 (0.95–1.29)	0.44697710
N06AX11 (mirtazapine)	0.98 (0.95–1.00)	0.20932285	0.97 (0.95–0.99)	0.15669208
N06AX12 (bupropion)	1.25 (1.00–1.56)	0.14624678	1.23 (0.99–1.53)	0.20065496
N06AX16 (venlafaxine)	1.08 (1.02–1.14)	0.02244824	1.08 (1.02–1.13)	0.03428351
N06AX17 (milnacipran)	0.95 (0.71–1.28)	0.84789139	0.95 (0.70–1.27)	0.92995513
N06AX18 (reboxetine)	0.88 (0.61–1.26)	0.71531554	0.88 (0.61–1.26)	0.77429725
N06AX21 (duloxetine)	1.11 (1.03–1.19)	0.02244824	1.10 (1.03–1.18)	0.03699542
N06AX22 (agomelatine)	0.87 (0.43–1.76)	0.84017664	0.86 (0.43–1.73)	0.92438759
N06AX26 (vortioxetine)	1.05 (0.38–2.92)	0.90623117	1.02 (0.37–2.86)	0.98690232
N06BX03 (piracetam)	1.29 (0.73–2.28)	0.61465795	1.28 (0.73–2.26)	0.68457110
N06CA01 (amitriptyline and psycholeptics)	1.04 (0.99–1.08)	0.17207707	1.04 (0.99–1.08)	0.25040741
N06DA02 (donepezil)	0.84 (0.80–0.88)	0.00000000	0.84 (0.80–0.88)	0.00000000
N06DA03 (rivastigmine)	0.81 (0.76–0.86)	0.00000000	0.81 (0.76–0.86)	0.00000000
N06DA04 (galantamine)	0.81 (0.75–0.88)	0.00000751	0.81 (0.75–0.88)	0.00000782
N06DX01 (memantine)	0.71 (0.67–0.75)	0.00000000	0.71 (0.67–0.75)	0.00000000
N07AA02 (pyridostigmine)	0.97 (0.80–1.17)	0.84789139	0.97 (0.80–1.18)	0.92995513
N07AA03 (distigmine)	0.83 (0.37–1.84)	0.82368734	0.82 (0.37–1.82)	0.87347840
N07AB01 (carbachol)	0.78 (0.38–1.58)	0.71531554	0.78 (0.38–1.58)	0.78758450
N07BA03 (varenicline)	2.12 (0.94–4.76)	0.18643186	2.06 (0.92–4.63)	0.23187851
N07BB01 (disulfiram)	1.32 (0.97–1.79)	0.20079331	1.30 (0.95–1.76)	0.25499043
N07BC02 (methadone)	1.01 (0.73–1.41)	0.90623117	0.99 (0.71–1.38)	0.98690232
N07CA01 (betahistine)	1.01 (0.97–1.06)	0.77549678	1.01 (0.97–1.06)	0.86480247

*Note*: The analysis was unadjusted and adjusted for socio‐economic groups giving odds ratios (ORs) with 95% confidence intervals (CIs) with *q* values based on conditional logistic regression analyses of nested case–control design.

### 3.5 Volcano plot analysis

**FIGURE 2:**
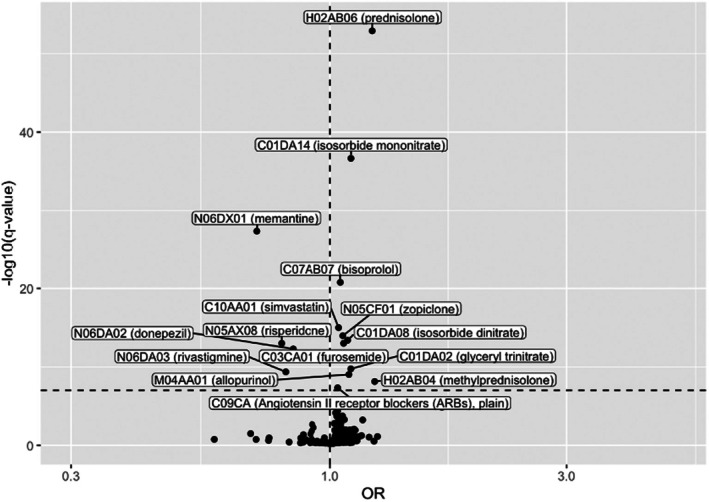
Volcano plot displaying results for the associations between studied drug categories and surgically treated cataract. Each solid circle represents a different drug. The x‐axis represents the natural log of the odds ratio (OR), and the y‐axis displays the *Q* values. The black vertical line indicates an OR = 1 (no association). The horizontal line indicates *Q* value for the associations on a logarithmic scale (–log10P). Solid circles indicate nonsignificant medications and labeled solid circles with ATC codes indicate significant medications in the model. No DM population, trend. Dichotomic, no adjustment for socioeconomic factors.

Next, we performed volcano plot analysis displaying results for the associations between studied systemic drug categories and surgically treated cataract (Figure 2).

## 4 DISCUSSION

It has been suggested that the use of systemic medication, not only systemic diseases, may alter normal lens physiology and lead to the development of cataract. We aimed to clarify the association between exposure to common systemic drug classes and the incident cataract surgeries in Finland in a longitudinal population‐based cohort study with a long follow‐up, covering non‐diabetic individuals.

Our study found that many systemic drugs showed some evidence for an association with an increased risk of surgically treated cataract. Noteworthy, the use of vasoactive agents such as ACE inhibitor ramipril, AT2R antagonist losartan, beta blocker bisoprolol, calcium channel blockers amlodipine and diltiazem, cardiovascular agents isosorbide mononitrate and isosorbide dinitrate, diuretic furosemide, statins simvastatin and atorvastatin, corticosteroid prednisolone, metabolic agent allopurinol, hormones testosterone and estradiol as well as psychoactive zopiclone was significantly associated with surgically treated cataract.

### 4.1 Hypertensive medication

Our nested case–control study design suggested that use of the calcium channel blocker amlodipine could be associated with an increased incidence of cataract. Our previous results associate amlodipine use with increased incidence of wet age‐related macular degeneration and diabetic macular oedema (Loukovaara et al., 2022; Loukovaara et al. 2024). Our results agree with recent findings suggesting the association between calcium channel blocker (and loop diuretics) and surgically treated cataract (Deng et al., 2023).

In our study, ramipril, inhibiting ACE in the renin–angiotensin system, and AT2R antagonist losartan, showed to be associated with the incidence of cataract surgery, confirming recent experimental findings (Choudhary et al., 2021). Choudhary et al. evaluated the role of the renin‐angiotensin system (RAS) in angiotensin II (AII)‐induced cataract formation in experimental hypertensive rats (Choudhary et al., 2021). Their results showed that the ocular RAS exacerbated the lenticular oxidative stress that may lead to cataract formation, suggesting that the RAS has an independent and important role in cataract formation under hypertensive conditions. However, in experimental conditions, olmesartan inhibited the risk of cataract formation in the hypertensive state via restoration of lenticular oxidative stress, ATPase function and ionic contents in the lens (Choudhary & Bodakhe, 2016).

### 4.2 Cardiovascular agents

Nitroglycerin (glyceryl trinitrate) has been used to treat angina pectoris, myocardial infarction and heart failure for long, acting as one of the main therapies for the common cardiac conditions. Therapeutic use of glyceryl trinitrate has been shown to produce mitochondrial reactive oxygen and reactive nitrogen species that have been implicated as causes of cataracts (Fang, Huang, et al., 2019; Fang, Ye, et al., 2019; Truscott, 2005). Treatment of cardiac patients with nitroglycerin has been shown to produce an imbalance in the systemic redox status, leading to the development of cataracts (El‐Gharabawy et al., 2016). Hypertensive patients were shown to have higher nitrite levels in the cataractous lens, showing a possible role of nitric oxide in the pathogenesis of human cataract (Ornek et al., 2003). In the light of the present study, it is possible that not only glyceryl trinitrate but also isosorbide mono‐ and dinitrate associate with cataract formation.

### 4.3 Beta blockers

Beta blockers have long been used for the treatment of cardiac arrhythmias. However, differences exist between the chemical structure, pharmacokinetic and pharmacodynamic properties. Current evidence supports the use of bisoprolol for the treatment of supraventricular arrhythmias, especially for atrial fibrillation (Muresan et al., 2022). Based on our analysis, especially the use of bisoprolol seems to associate with incident cataract surgery. However, other beta blockers (metoprolol, atenolol) might also be involved with cataract formation. Binding of the drug to ocular tissue (lens proteins, melanin, DNA) may produce phototoxic side effects in the eye (Roberts, 2002). According to the literature, a series of drugs, for example, beta blockers exhibit melanin‐binding properties that could relate to cataract development (Steiner et al., 1990).

### 4.4 Hyperlipidemia medication

Mechanism by which statins are associated with cataract formation is biologically possible. Human crystalline lens requires high cholesterol for proper membrane formation and maintenance of transparency. In fact, lens membrane contains the highest cholesterol content compared to any other membrane (Cenedella, 1996). Thus, the use of statins, which inhibit lens cholesterol biosynthesis, can be associated with cataracts both in animals and humans.

Our data confirm the findings of a recent study investigating the association between statin exposure and the incident cataract risk, showing that statin use could contribute to rising rates of cataract surgery (Erie et al., 2016). Our data showed an association with simvastatin and atorvastatin use with surgically treated cataract, but, for example, pravastatin and rosuvastatin did not show a strong association.

### 4.5 Corticosteroids

Corticosteroid exposure and risk of cataract has been studied for decades. Indeed, the first study showing that long‐term use of systemic corticosteroids may be associated with the development of cataract was published six decades ago (Sundmark, 1963). Recent meta‐analysis showed that not only systemic but also inhaled corticosteroids may increase the risk of cataract in patients with both chronic obstructive pulmonary disease and asthma (Savran & Suppli Ulrik, 2023). In addition to cataract, both short‐ and long‐term corticosteroid treatment may induce various other adverse events, including Cushing syndrome, hypertension, diabetes, and glaucoma.

### 4.6 Hormones

Regarding exogenous oestrogen, conflicting data exist for the use of postmenopausal hormone therapy (Defay et al., 2003; Zetterberg & Celojevic, 2015). Very recently, sex hormone combinations were shown to reduce the risk of surgically treated cataract (Deng et al., 2023). In our longitudinal study, the use of testosterone increased the incidence of surgically treated cataract by 63% (3–155%) and the use of estradiol by 13% (3–24%).

### 4.7 Allopurinol

Our findings suggesting that allopurinol could increase the incidence of cataract agree with earlier findings (Fraunfelder & Meyer, 1984). Cumulative doses of over 400 g or continuous use for over 3 years have been associated with increased risk of cataract surgery (Garbe et al., 1998).

### 4.8 Insomnia medication

Insomnia is a known risk factor for mental health disorders (depression, anxiety, alcohol dependence), metabolic syndrome, hypertension and coronary heart disease, worsened quality of life and increased mortality (De Crescenzo et al., 2022). Pharmacotherapy is commonly used to treat insomnia. Medications for the treatment of chronic insomnia include several different drug classes, such as benzodiazepine, non‐benzodiazepine gamma‐aminobutyric acid (GABA)‐A modulators, dual orexin receptor antagonists, melatonin receptor agonists and histamine antagonists. Our report is the first one associating use of zopiclone with the potential risk of incident cataract surgery. Chronic insomnia is a severe disorder, with long‐term serious impacts on health.

### 4.9 Neuropsychiatric medications

In our study, patients on memantine or donepezil used for Alzheimer disease, rivastigmine for Alzheimer or Parkinson disease, and risperidone for schizophrenia or psychosis were operated for cataract less often than patients without these medications. The reason for this may be delays in patients seeking help with their visual problems, also delaying the diagnosis of cataract. Severe cataract is clinically more common in the neuropsychiatric patient groups, suggesting these patients may wait longer with cataracts before it is diagnosed. In previous literature, schizophrenia per se was not found to be associated with an increased risk of cataract formation, unlike use of antipsychotics chlorpromazine and prochlorperazine (Ruigómez et al., 2000). Interestingly, in the study by Ruigomez et al., the incidence rate of cataract was lower in the schizophrenic group. In another study, second‐generation antipsychotics, such as risperidone, were associated with a higher risk of cataract than first‐generation antipsychotics, even when adjusted for type 2 diabetes or hyperlipidaemia (Fang, Huang, et al., 2019; Fang, Ye, et al., 2019). Periodic ophthalmological examination would be advisable in these patients even without complaints of visual symptoms. Of note, for two decades the use of memantine has been shown to associate with increased incidence of cataracts (Rossom & Adityanjee Dysken, 2004). A recent case–control study showed no association between selective serotonin reuptake inhibitors or other antidepressants and cataract formation (Becker et al., 2020).

### 4.10 Strengths and limitations

Strength of this longitudinal study is a relatively large and genetically homogenous study population with data on systemic medications and socio‐economic background. However, regarding socio‐economic background no established definition exists (Oakes & Rossi, 2003). In addition, our study findings were compared with previous publications. Nevertheless, there are limitations that we could not overcome, including the retrospective nature and the design that was limited to surgical coding data and redeemed prescriptions.

Most historical studies on medication exposures use general outcomes and broad drug classes that could mask harmful effects. Ideally, we should look at pairs of individual medications with individual outcomes (Sendor & Stürmer, 2022). Interpretation of our results must be extremely cautious, since despite adjusting, some residual confounding factors could persist in the analysis. Due to our study design, we had no information of environmental or lifestyle factors (such as smoking or alcohol consumption, diet, ionizing radiation exposure or exercise) or other modifiable factors that could impact the development of age‐related cataract formation.

In addition, the severity of the underlying systemic diseases was not considered. Neither was the exact type of cataract known (age‐related nuclear cataract, posterior subcapsular, cortical or mixed cataract). Of note, a previous meta‐analysis showed an association between hypertension and posterior subcapsular cataract, but not with nuclear cataract (Yu et al. 2014). In addition, in our study, we had no way of analysing daily dose of medication or dose‐dependent effects. Therefore, some findings could be due to confounders. However, our nested case–control design was adjusted for potential confounders such as socioeconomic factors aiming to reduce bias. In pharmacoepidemiology, there is always a risk for indication bias. Thus, it remains unclear if the associations between studied systemic drugs and the development of cataract are causal (i.e., incident cataract surgery). Despite the large data set consisting of 106 127 individuals, the causality is not always clear. We cannot be sure if it is the medication that causes cataract or if the pathogenesis of cataract is due to the different severity of background diseases. The biological effects of systemic drugs in patients using multiple drugs may also cause confounding. Many drugs are often co‐prescribed, making interpretation of the results challenging. A drug may look harmful based on statistical analyses because of the outcome (i.e., need for cataract surgery). Study outcomes are known to depend on treatment compliance, discontinuation, continuous use, dosing, monotherapy, polytherapy and switching of medication.

A principle of the Finnish public healthcare system is to offer all citizens equal medical treatment. However, there can be heterogeneity, especially concerning the local resources to operate the cataract in Finland despite nationwide treatment guidelines and recommendations (Finnish Current Care Guideline).

Since decades, it has been known that many commonly used drugs in individuals over 50 years may predispose to the development of cataract. It is known that common systemic diseases (such as hypertension, dyslipidaemia, cardiovascular and respiratory diseases) may increase the risk of cataract per se, not only the drugs. Additionally, systemic drugs may have larger relative effects in sicker populations (Guyatt et al., 2008). Variability related to study results may arise from differences in study settings and populations (Deng et al., 2023). Our study was conducted in a Finnish, predominately Caucasian population in the Northern hemisphere with less myopia than in, for example, Asian populations. Our data did not include axial length or refraction. In the future, it would be interesting to study whether myopia, which in itself increases the risk for cataract, modifies the association between systemic medications and cataract or not.

To conclude, age‐related cataract continues to be a significant eye disease worldwide. Our study findings, suggesting that systemic therapy may increase the incidence of cataract surgery, warrant further investigation. It is likely that some associations are due to confounders, but biochemical effects capable of causing or accelerating cataract formation have been reported for many of the studied substances. We may report significant findings of drug exposure in cataract development even without true associations. In the future, signal detection methods could probably be used to scan through all possible medication‐outcome pairs to find signals, that is, pairs where more events have occurred than expected by chance.

## AUTHOR CONTRIBUTIONS

All authors have given their final approval of the version to be published; AR, SL, JH and TL have given the agreement to be accountable for all aspects of the work in ensuring that questions related to the accuracy or integrity of any part of the work are appropriately investigated and resolved. JH had full access to all the data in the study and takes responsibility for the integrity of the data and the accuracy of the data analysis. Concept and design: SL, AR, JH. Acquisition (JH), analysis (JH) or interpretation of data (AR, JH, TL, SL). Drafting of the manuscript: SL, AR, JH, TL. Critical revision of the manuscript for important intellectual content: SL, JH, TL, AR.

## CONFLICT OF INTEREST STATEMENT

No potential conflict of interest relevant to this report.

## Supporting information


**Figure S1.** Causal graph used to select adjustment set for analyses (https://dagitty.net/dags.html?id=f8dPZkxW).
