# Supplementary material for: Systemic medications associate with surgically treated cataract among adults over 50 years in Finland
Source: Acta Ophthalmol. 2026 Mar 11;104(6):e630–47. doi: 10.1111/aos.70118 (PMC13460381; doi:10.1111/aos.70118)
Supplement: Supplementary file 1 [file AOS-104-e630-s001.docx]

Supplemental Figure 1. Causal graph used to select adjustment set for analyses

(https://dagitty.net/dags.html?id=f8dPZkxW).
